# Causal relationships between atopic dermatitis and psychiatric disorders: a bidirectional two-sample Mendelian randomization study

**DOI:** 10.1186/s12888-023-05478-1

**Published:** 2024-01-03

**Authors:** Suqi Cao, Zicheng Zhang, Lei Liu, Yin Li, Wei Li, Yunling Li, Dingfeng Wu

**Affiliations:** 1 National Center, Children’s Hospital, Zhejiang University School of Medicine, National Clinical Research Center for Child Health, Hangzhou, 310052 People’s Republic of China; 2 School of Biomedical Engineering, School of Information and Communication Engineering, Hainan University, Haikou, 570228 People’s Republic of China; 3 National Clinical Research Center for Ocular Diseases, Eye Hospital, Wenzhou Medical University, Wenzhou, 325027 People’s Republic of China; 4 Hainan Institute of Real World Data, Haikou, 570228 People’s Republic of China; 5 Department of Gastroenterology, School of Life Sciences and Technology, The Shanghai Tenth People’s Hospital, Tongji University, Shanghai, 200072 People’s Republic of China; 6 Department of Dermatology, Children’s Hospital, Zhejiang University School of Medicine, National Clinical Research Center for Child Health, Hangzhou, 310053 People’s Republic of China

**Keywords:** Atopic dermatitis, Autism spectrum disorder, Psychiatric disorder, Human genetics, Causal pathways

## Abstract

**Background:**

Observational studies have suggested the potential associations between atopic dermatitis (AD) and psychiatric disorders. However, the causal relationship between them remains uncertain. This study aimed to evaluate the potential bidirectional causal relationship between AD and psychiatric disorders, including autism spectrum disorder (ASD), major depressive disorder (MDD), attention deficit hyperactivity disorder (ADHD), bipolar disorder (BD), anorexia nervosa (AN), Tourette syndrome (TS), schizophrenia, and anxiety.

**Methods:**

Bidirectional two-sample Mendelian randomization (MR) was employed to elucidate the causality between AD and psychiatric disorders, using summary statistics from the most comprehensive genome-wide association studies conducted on AD (N_cases_ = 60,653, N_controls_ = 804,329). Psychiatric disorders were derived from the Psychiatric Genomics Consortium and were independent of AD data sources. The MR analysis entailed the implementation of multiple methods, including the inverse variance weighted method, MR-Egger regression method, weighted median method, simple mode method, and weighted mode method.

**Results:**

Bidirectional two-sample MR analysis uncovered significant causal associations between AD and severe psychiatric disorders. Specifically, liability to AD was associated with increased risk of ADHD (OR = 1.116; 95% CI: [1.009, 1.234]; *P* = 0.033) and ASD (OR = 1.131; 95% CI: [1.023, 1.251]; *P* = 0.016). Additionally, evidence suggested that liability to ADHD (OR = 1.112; 95% CI: [1.094, 1.130]; *P* = 9.20e-40), liability to AN (OR = 1.1; 95% CI: [1.068, 1.134]; *P* = 4.45e-10) and liability to BD (OR = 1.067; 95% CI: [1.009, 1.128]; *P* = 0.023) were associated with an increased risk of AD. Only the causal association between AD and ASD was independent of the reverse effect bias. These causal associations were robust and not affected by biases of heterogeneity and horizontal pleiotropy.

**Conclusions:**

Our study emphasizes the significant causal association between AD and an increased risk of ASD, and also identifying BD and AN as risk factors for AD.

**Supplementary Information:**

The online version contains supplementary material available at 10.1186/s12888-023-05478-1.

## Introduction

Psychiatric disorders exhibit a high prevalence, affecting approximately 970 million individuals worldwide in 2019, as reported by the World Health Organization [1]. These disorders often manifest with clinically significant impairments in cognition, emotion regulation, or behavior, which can culminate in overt suicidal tendencies [2]. Regrettably, the underlying causes or etiology of mental disorders frequently remains elusive and uncertain. Likewise, atopic dermatitis (AD) is a prevailing chronic inflammatory skin disorder [3]. AD displays a notable degree of heterogeneity in clinical features, disease severity, and its overall course, making it challenging to establish a gold standard for clinical diagnosis. Nevertheless, essential clinical manifestations remain consistent, including the presence of eczematous skin lesions, intense pruritus, and a disease course characterized by either chronicity or recurrent episodes [4]. The prevalence and incidence of AD have exhibited an escalating trend over the past few decades. As a result, AD has become the 15th most prevalent non-pathogenic dermatitis and the skin disease burden with the utmost impact [5]. Numerous studies have established a correlation between AD and various comorbidities. Particularly, the association between AD and psychiatric disorders has attracted significant interest, as it explores the biological mechanisms linking the hypothalamic–pituitary–adrenal (HPA) axis and sympathetic nervous system (SNS) to chronic inflammatory diseases and mood disorders [6]. However, the causative relationship between these two diseases remains elusive.

The etiology of AD is believed to entail a multifaceted interplay between genetic predisposition, environmental factors, perturbed immune function, and psychological influences [7]. Studies have indicated that psychological stress triggers the upregulation of neuropeptide mediators in the brain, endocrine organs, and peripheral nervous system, directly impacting immune cells and resident cells in the skin, thus culminating in the clinical manifestation of AD [8]. Furthermore, compelling evidence suggests that intense pruritus, heightened rates of sleep disturbances, societal stigmatization, social isolation, compromised quality of life, and neuroinflammation may amplify the susceptibility to anxiety, depression, and suicidality among individuals with AD [9]. Meta-analyses have also shed light on the intricate relationship between the neurological or psychiatric disorders and AD. For instance, comorbidity between AD and autism spectrum disorder (ASD) has been identified [10], with ASD patients exhibiting an elevated risk of developing AD, and vice versa [11]. Similarly, children with AD demonstrate an increased likelihood of attention deficit hyperactivity disorder (ADHD) [12]. However, certain questionnaires reported no significant disparity in ADHD scores between the AD group and control group [13]. Depression, suicidality, parental depression, and augmented antidepressant usage have been associated with AD [14], albeit some studies yield conflicting outcomes [15]. Given that both psychiatric disorders and AD encompass ambiguous and subtle onsets, and the associations delineated by prior research may partially stem from reverse causation and/or residual confounding effects commonly encountered in observational studies, disentangling the chronological sequence of onset and causality between these conditions poses a formidable challenge.

In this particular context, Mendelian randomization (MR) emerges as a valuable tool for inferring causality by exploring associations between diverse diseases. MR leverages genetic variations identified through genome-wide association studies (GWAS) as instrumental variables for exposure, enabling the estimation of causal relationships between exposure and outcome [16]. More recently, a two-sample Mendelian analysis employing meta-analysis data from the GWAS conducted by Paternoster et al. in 2015 failed to establish any association between AD and depression or anxiety [17]. However, it is important to note that these data are constrained and lacks the latest GWAS data. Moreover, the aforementioned study solely explored the relationship between AD and depression as well as anxiety. Therefore, the objective of this investigation aimed to evaluate the potential bidirectional causal relationship between AD and psychiatric disorders, including autism spectrum disorder (ASD), major depressive disorder (MDD), ADHD, bipolar disorder (BD), anorexia nervosa (AN), Tourette syndrome (TS), schizophrenia, and anxiety, through a two-sample Mendelian randomization study design employing the most up-to-date comprehensive GWAS data.

## Methods

### Data sources

#### Atopic dermatitis

The GWAS large-scale summary statistic profile (SNPs) of atopic dermatitis was obtained from Ashley et al. GWAS analyses (N_cases_ = 60,653; N_controls_ = 804,329) using the individuals of European ancestry [18].

#### Psychiatric disorders

We obtained a comprehensive set of eight psychiatric disorders, including autism spectrum disorder (ASD) [19], major depressive disorder (MDD) [20], attention deficit hyperactivity disorder (ADHD) [21], bipolar disorder (BD) [22], anorexia nervosa (AN) [23], Tourette syndrome (TS) [24], schizophrenia [25] and anxiety [26], from the publicly available databases. Five GWAS summary statistic profiles mentioned above, including ADHD, BD, AN, TS and anxiety, were directly downloaded from the Psychiatric Genomics Consortium (PGC, https://www.med.unc.edu/pgc/download-results), and the others, including ASD, MDD and SCZ, were obtained from the IEU OpenGWAS project (https://gwas.mrcieu.ac.uk/, updated to 2023–03-20). The sample sizes for these eight psychiatric disorders varied from 3,833 to 173,005 individuals of European ancestry.

The detailed information on AD and psychiatric disorders was shown in Table 1. To minimize the risk of inflated Type 1 error rates, there were few overlapping participants between the exposure and outcome groups in the two-sample Mendelian randomization (MR) analysis.

**Table 1:** Overview of the source of data

GWAS ID/Source	Year	Trail	Consortium	Sample size	Population	Build	Download link
GCST90244787	2023	Atopic Dermatitis	Ashley et al.[18]	60,653 cases; 804,329 controls	European	HG19/GRCh37; HG19/GRCh38	https://www.ebi.ac.uk/gwas/studies/GCST90244787
ieu-a-1185	2017	Autism Spectrum Disorder	PGC	18,382 cases; 27,969 controls	European	HG19/GRCh37	https://gwas.mrcieu.ac.uk/datasets/ieu-a-1185/
ieu-a-1188	2018	Major Depressive Disorder	PGC	59,851 cases; 113,154 controls	European	HG19/GRCh37	https://gwas.mrcieu.ac.uk/datasets/ieu-a-1188/
PGC-adhd2019	2019	Attention Deficit Hyperactivity Disorder	PGC	19,099 cases; 34,194 controls	European	HG19/GRCh37	https://figshare.com/ndownloader/files/28169253/daner_adhd_meta_filtered_NA_iPSYCH23_PGC11_sigPCs_woSEX_2ell6sd_EUR_Neff_70.meta.gz
PGC-bip2019	2019	Biopolar Disorder	PGC	20,352 cases; 31,358 controls	European	HG19/GRCh37	https://figshare.com/ndownloader/files/28169307/daner_PGC_BIP32b_mds7a_0416a.gz
PGC-an2019	2019	Anorexia Nervosa	PGC	768 cases; 3,065 controls	European	HG19/GRCh37	https://figshare.com/ndownloader/files/28169271/pgcAN2.2019-07.vcf.tsv.gz
PGC-ts2019	2019	Tourette Syndrome	PGC	4,819 cases; 9,488 controls	European	HG19/GRCh37	https://figshare.com/ndownloader/files/28169940/TS_Oct2018.gz
ieu-b-5102	2022	Schizophrenia	PGC	52,017 cases; 75,889 controls	European	HG19/GRCh37	https://gwas.mrcieu.ac.uk/datasets/ieu-b-5102/
PGC-anx2016	2016	Anxiety	PGC	7,016 cases; 14,745controls	European	HG19/GRCh37	https://figshare.com/ndownloader/files/28570812/anxiety.meta.full.cc.tbl.gz
ebi-a-GCST90013474	2021	Sex (age adjusted)	EBI	245,351 cases; 206,951 controls	European	HG19/GRCh37	https://gwas.mrcieu.ac.uk/datasets/ebi-a-GCST90013474/
ukb-b-3018	2018	Age at death	UKBiobank	11,856 samples	European	HG19/GRCh37	https://gwas.mrcieu.ac.uk/datasets/ukb-b-3018/
ebi-a-GCST001475	2012	Obesity	EBI	5,530 cases; 8,318 controls	European	HG19/GRCh37	https://gwas.mrcieu.ac.uk/datasets/ebi-a-GCST001475/
ukb-b-16499	2018	Non-cancer illness code, hayfever/allergic rhinitis	UKBiobank	26,107 cases; 436,826 controls	European	HG19/GRCh37	https://gwas.mrcieu.ac.uk/datasets/ukb-b-16499/
ebi-a-GCST90014325	2021	Asthma	EBI	56,167 cases; 352,255 controls	European	HG19/GRCh37	https://gwas.mrcieu.ac.uk/datasets/ebi-a-GCST90014325/
finn-b-I9_CEREBVASC	2021	Cerebrovascular diseases	FinnGen biobank	15,724 cases; 203,068 controls	European	HG19/GRCh37	https://gwas.mrcieu.ac.uk/datasets/finn-b-I9_CEREBVASC/

*PGC* Psychiatric Genomics Consortium, *EBI* EMBL’s European Bioinformatics Institute

### Selection of instrumental variable using SNPs

**Fig. 1:**
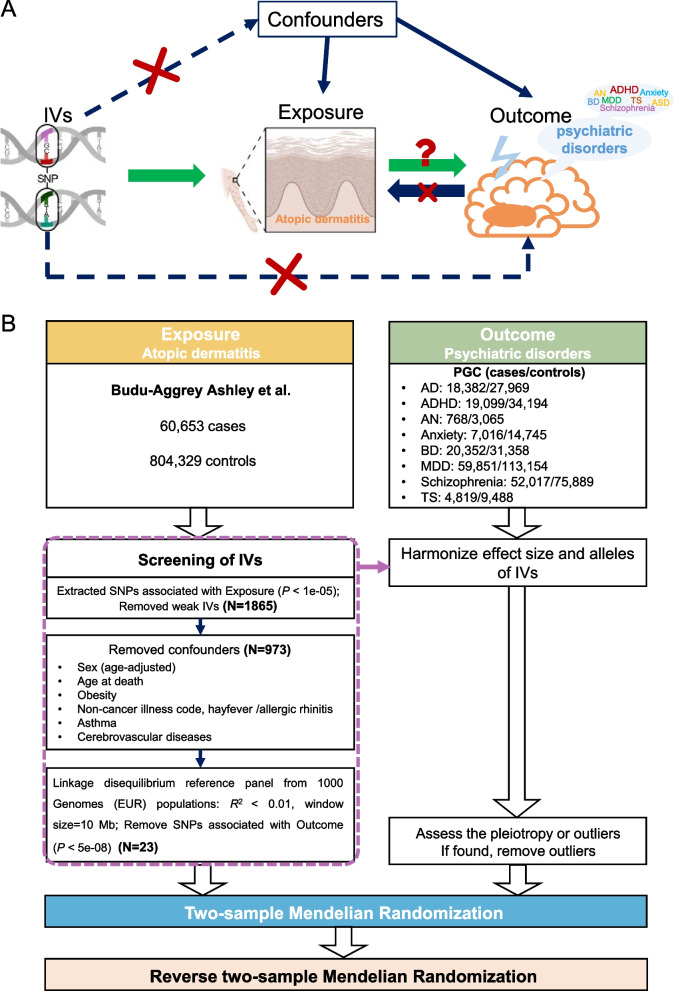
Study assumption and workflow of the two-sample MR analysis between atopic dermatitis and psychiatric disorders

To conform to the standard MR analysis, we used SNPs as instrumental variables (IVs) to establish the causal relationship between exposure and outcome [27], according to the three assumptions shown in Fig. 1A: (1) direct correlation between the IVs and the exposure; (2) absence of direct association between the IVs and confounding factors; and (3) absence of direct association between the IVs and outcomes. To meet the above assumptions, a synthesis flow chart is shown in Fig. 1B. The results from the largest AD GWAS to date from a 40-item cohort were used to generate the IVs [18]. SNPs associated with each exposure at the genome-wide significance level of *P* < 1e-05 were selected as potential IVs, and any weak IVs defined as *F-statistic* ≤ 50 using the formula ${\text{F}}=\frac{{Beta}^{2}}{{SE}^{2}}$ were excluded. Subsequently, we obtained confounding SNPs from the EBI database, UK biobank and FinnGen biobank, including biological sex, age at death, obesity, hayfever/allergic rhinitis, asthma and cerebrovascular disease. All confounding SNPs were excluded to mitigate the effect of horizontal pleiotropy. To ensure that SNPs were independent, we pruned the variants by linkage disequilibrium (R^2^ threshold = 0.01, window size = 10 Mb) on the basis of the 1,000 Genomes European reference panel. To ensure that there was no direct association between IVs and outcome, we excluded those IVs that showed a direct correlation with outcome (*P* < 5e-08). The effect estimates of both exposure and outcome variants were harmonized and expressed per effect allele increase, and possible palindromic SNPs were excluded. Furthermore, we applied the MR pleiotropy residual sum and Outlier test to detect and exclude any horizontal pleiotropic SNPs [28].

### Two-sample mendelian randomization analysis

#### Forward two-sample Mendelian randomization analysis

Two-sample MR analysis was conducted to investigate the causal association between the exposure (AD) and outcome (psychiatric disorders) variables, while minimizing the influence of potential confounders. Based on the selection of available IVs, five widely accepted MR approaches were performed in our study including inverse variance weighted (IVW) method [29], MR-Egger regression method [30], weighted median method [31], simple mode method [32], and weighted mode method [31]. Additionally, for a supplementary analysis, we introduced a novel MR method founded on constrained maximum likelihood (cML) and model averaging (MA), called cML-MA [33]. Specifically, IVW method can provide accurate estimate when there was an absence of heterogeneity and horizontal pleiotropy among IVs. Weighted Median method was chosen for MR analysis when there was heterogeneity but no horizontal pleiotropy. In cases where horizontal pleiotropy was present, the MR-Egger regression method was employed to detect and addressed pleiotropy. The Simple Mode method provided robustness for pleiotropy, while the Weighted Mode method was more sensitive to the selection of bandwidth for mode estimation. We also performed the cML-MA method, which can control Type I errors with high power. The threshold of *P* < 0.05 was considered to indicate a significant causal association.

#### Reverse two-sample Mendelian randomization analysis

The reverse two-sample MR analysis treating psychiatric disorders as the exposure and AD as the outcome investigated the insignificant causal association, indicating the unidirectionality of the causal effect. The specific process and threshold followed the aforementioned forward two-sample MR analysis.

#### Sensitivity analysis

In our study, the IVW, MR-Egger, weighted median, simple mode and weighted mode methods demonstrated similar magnitudes and directions of the causal effects. The MR-Egger regression intercept method was performed to identify the horizontal pleiotropy. Both the MR-Egger regression method and IVW method were utilized to examine the existence of heterogeneity using Cochran Q statistics. The IVW outlier method and MR-Egger regression outlier method were used to remove outliers from the results. The leave-one-out method was conducted to assess the influence of individual SNPs on the causal signal and determine whether the causal associations were driven by any specific SNP. The threshold of *P* > 0.05 was defined to indicate the absence of heterogeneity and horizontal pleiotropy.

### Statistical analysis

All analyses were performed using the 'TwoSampleMR' (v0.5.6), 'MRPRESSO' (v1.0), and 'MRcML' (v0.0.0.9) R packages within the R software (v4.2.3) equipped on the Rstudio (v1.4.1106) platform. In the absence of evidence suggesting pleiotropy among the selected IVs, indicated by an MR-Egger intercept *P* > 0.05, the IVW method emerges as the most reliable approach. To improve the visualization of the IVW and MR-Egger estimates, we also implemented the IVW and MR-Egger radial variation models in the 'RadialMR' (v1.0) R package.

## Results

### Identification of IVs

Referring to rigorous quality control measures and adherence to standard protocols (details as shown in Methods), a total of 23 SNPs (Table 2) were selected as IVs for the analysis. Briefly, in the SNP selection process, we initially considered 11,164 SNPs associated with the AD (genome-wide significance level of *P* < 1e-05) as potential IVs. Then 9,299 weak IVs (*F-statistic* ≤ 50) were excluded. Further refinement involved the removal of 892 IVs that were confounded by factors such as biological sex, age at death, obesity, hayfever/allergic rhinitis, asthma and cerebrovascular disease. Finally, we eliminated 950 IVs that were either non-independent (R^2^ threshold = 0.01, window size = 10 Mb) or direct association with the outcomes (*P* < 5e-08). After removing the pleiotropic IVs identified by the IVW outlier method and MR-Egger regression outlier method (Figure S1), there was no obvious evidence of horizontal pleiotropy of these IVs (*P* values in the MR-Egger regression outlier method were greater than 0.05).

**Table 2:** The instrumental variables of atopic dermatitis (significance level of *P* < 1e-05)

Selected SNPs	EA/non-EA	Beta	SE	F-value	*P*-value
rs75360998	A/G	0.263	0.031	70.858	4.04E-17
rs192129010	C/G	0.174	0.020	77.852	1.17E-18
rs10443207	C/A	-0.077	0.008	93.998	3.36E-22
rs4845779	C/T	-0.096	0.007	177.503	1.87E-40
rs61801951	T/C	0.148	0.018	68.380	1.42E-16
rs12133641	G/A	0.065	0.007	90.761	1.72E-21
rs2859274	C/T	-0.059	0.008	58.229	2.45E-14
rs11811788	G/C	0.065	0.008	72.407	1.85E-17
rs2272128	A/G	-0.095	0.008	151.672	8.14E-35
rs41293876	C/G	-0.103	0.013	65.222	7.02E-16
rs6996614	A/C	0.064	0.008	69.391	8.48E-17
rs61839660	T/C	0.114	0.012	85.369	2.63E-20
rs10822037	C/T	0.060	0.007	78.487	8.53E-19
rs28520436	T/C	0.180	0.018	105.140	1.22E-24
rs3814707	A/G	-0.088	0.008	134.063	5.71E-31
rs7127307	C/T	-0.055	0.007	68.562	1.29E-16
rs2227491	C/T	0.053	0.007	63.766	1.46E-15
rs2415269	A/G	-0.060	0.007	67.463	2.26E-16
rs7189563	A/C	-0.051	0.007	59.792	1.11E-14
rs17881320	T/G	0.087	0.012	52.155	5.34E-13
rs2967677	T/C	0.082	0.009	84.888	3.35E-20
rs2738783	G/T	0.090	0.009	107.120	4.49E-25
rs4821569	G/A	0.049	0.007	53.203	3.14E-13

*EA* Effect allele, *SE* Standard error

### The causal relationship of atopic dermatitis on psychiatric disorders

**Fig. 2:**
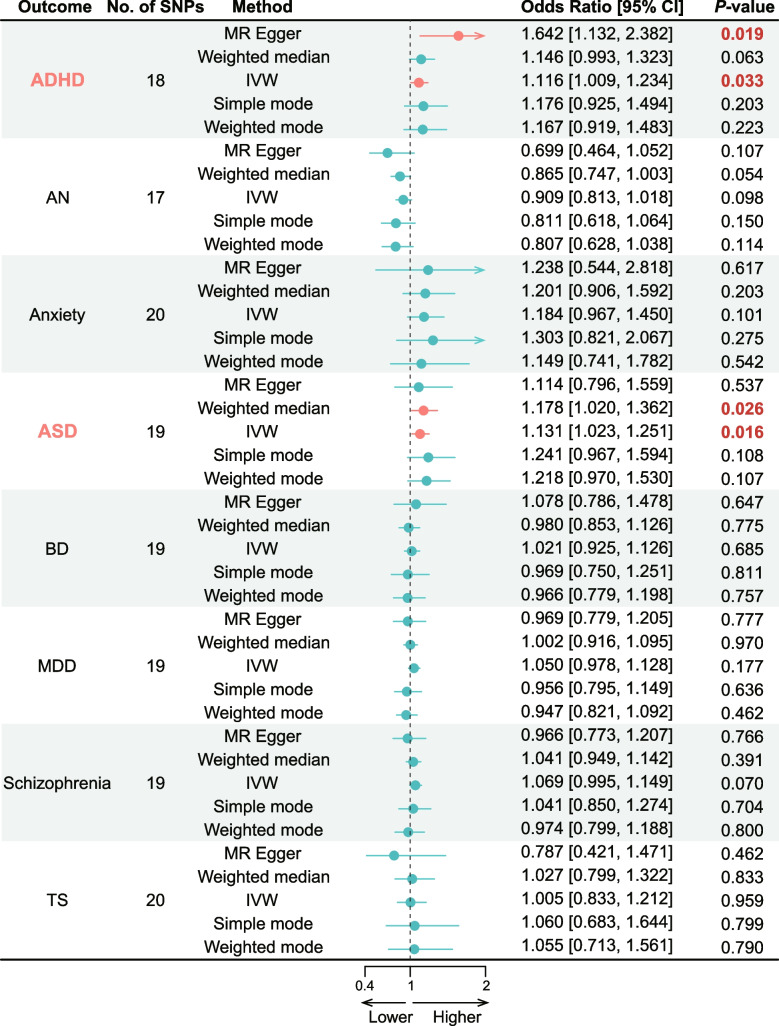
Forest plots of the causal association using two-sample MR analysis methods between atopic dermatitis and psychiatric disorders. Atopic dermatitis as exposure, and psychiatric disorder including ADHD, AN, Anxiety, ASD, BD, MDD, Schizophrenia and TS as outcomes

**Fig. 3:**
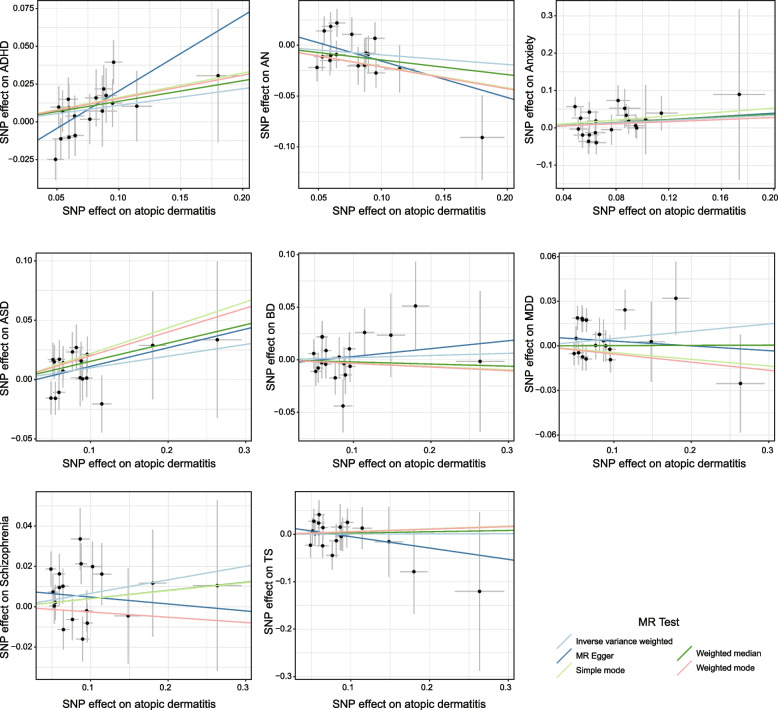
Scatter plots of the causal relationships between atopic dermatitis and psychiatric disorders using two-sample MR analysis methods. MR analysis includes inverse variance weighted, MR Egger, Simple mode, Weighted median and Weighted mode method

Regarding the psychiatric disorders investigated, there were significant causal association of AD on ADHD and ASD (Figs. 2 and 3). In ADHD, 18 IVs were available, and the IVW method revealed that AD was associated with the increased risk of ADHD (OR = 1.116; 95% CI: [1.009, 1.234]; *P* = 0.033, Fig. 2, Table S1), which was further verified by the MR-Egger regression method (OR = 1.642; 95% CI: [1.132, 2.382]; *P* = 0.003, Fig. 2, Table S1) in the same direction and causal effect. Simultaneously, the causal association between AD and ASD was also uncovered using two distinct MR methods with the aid of 19 IVs. The IVW method revealed an increased risk effect of AD to ASD (OR = 1.131; 95% CI: [1.023, 1.251]; *P* = 0.016, Fig. 2, Table S1), while the weighted median method affirmed the identical causal effect of AD on ASD (OR = 1.178; 95% CI: [1.020, 1.362]; *P* = 0.026, Fig. 2, Table S1). As a supplementary analysis, we employed the cML-MA method and observed that AD was associated with an increased risk of ASD (*P* = 0.018, Table S2).These findings demonstrated evidence supporting the causal association of AD on the increased risk of ADHD and ASD.

### Reverse MR analysis of psychiatric disorders on atopic dermatitis

To identify any reverse causal associations, we used psychiatric disorders as exposure and AD as outcome. In the screening of available IVs for reverse MR analysis, the three underlying assumptions outlined in the methodology must be satisfied. As shown in Table S3-S4 and Figure S2, the ADHD exhibited an increased risk effect to AD (OR = 1.112; 95% CI: [1.094, 1.130]; *P* = 9.20e-40), while the other four MR methods (weighted median, simple mode, weighted mode and cML-MA) affirmed the identical causal effect of ADHD on AD. Furthermore, we discovered an increased risk effect of AN to AD with the IVW method (OR = 1.1; 95% CI: [1.068, 1.134]; *P* = 4.45e-10). With the exception of MR-Egger, all other MR methods provided further support for the existence of the causal association of AN on AD (Table S3-S4 and Figure S2). Finally, the BD also displayed an increased risk effect to AD with IVW method (OR = 1.067; 95% CI: [1.009, 1.128]; *P* = 0.023) and cML-MA method (*P* = 0.024). The robustness of these findings was supported by the absence of horizontal pleiotropy and heterogeneity, as assessed through the MR-Egger regression method and Cochran's IVW Q test method (*P* > 0.05, Table S5). In conclusion, the causal association between AD and ASD was independent to the bias of reverse MR analysis result.

### Sensitivity analyses

To validate the causal association identified by the MR-Egger, weighted mode, simple mode, and weighted median methods from our bi-directional MR analysis, we performed sensitivity analyses using several methods, including the MR-Egger regression intercept method, Cochran's IVW Q test, Cochran's MR-Egger regression Q test, and the "leave-one-out" approach (Table 3, Table S5 and Figure S3). We found no evidence of horizontal pleiotropy between AD and psychiatric disorders, as indicated by *P* values greater than 0.05 obtained from the MR-Egger regression intercept method. IVW outlier analysis and MR-Egger outlier analysis excluded the outliers from the above results. The "leave-one-out" approach was employed to assess the robustness of the causal association and unaffected with the influence of individual SNPs on the above results. Furthermore, the results of the Cochran's IVW Q test and Cochran's MR-Egger regression Q test showed no significant heterogeneity with *P* > 0.05.

**Table 3:** The horizontal pleiotropy and heterogeneity results

Outcome	Horizontal pleiotropy			Heterogeneity			
				**MR Egger**		**IVW**	
	**Intercept**	**SE**	***P*-value**	**Q**	***P*-value**	**Q**	***P*-value**
ADHD	-0.029	0.014	0.0504	10.385	0.846	14.863	0.605
AN	0.020	0.015	0.210	17.236	0.305	19.209	0.258
Anxiety	-0.003	0.030	0.915	16.327	0.570	16.339	0.635
ASD	0.001	0.013	0.928	17.472	0.423	17.481	0.490
BD	-0.004	0.012	0.725	14.498	0.632	14.626	0.687
MDD	0.006	0.008	0.450	25.621	0.082	26.522	0.088
Schizophrenia	0.008	0.009	0.359	22.894	0.153	24.090	0.152
TS	0.019	0.024	0.432	9.671	0.942	10.318	0.945

*IVW* Inverse variance weighted, *SE* Standard error, *ADHD* Attention deficit hyperactivity disorder, *AN* Anorexia nervosa, *ASD* Autism spectrum disorder, *BD* Bipolar disorder, *MDD* Major depressive disorder, *TS* Tourette syndrome

## Discussion

In recent years, with the increasing availability of extensive GWAS summary statistics, MR methods have gained widespread usage in establishing causal relationships between co-occurring traits or diseases [16]. This method offers a cost-effective and rationalized strategy for clinical trials and opens new avenues for research design. Our primary objective was to investigate the potential causal relationship between AD (a prevalent chronic inflammatory skin disease) and psychiatric disorders (ADHD, ASD, AN, BD, MDD, TS, schizophrenia, and anxiety). To examine this relationship, we employed a bidirectional two-sample MR analysis. To minimize the influence of confounders and ensure the robustness of our results, we also carefully controlled for biological sex, age at death, obesity, hayfever/allergic rhinitis, asthma, and cerebrovascular disease. Given the superior power of the IVW method under specific conditions [29], our study's primary conclusion hinges on the IVW method, with other methods offering supplementary reinforcement.

In this study, we made a noteworthy discovery, indicating a nominally significant impact of genetically determined AD on the heightened risk of ASD among individuals of European ancestry. This discovery provides an answer to the previously ambiguous matter of the temporal order (i.e., causality) between the comorbidities of AD and ASD [10]. Additionally, this finding might support the hypothesis that neuroinflammation contributes to the pathogenesis of ASD by influencing brain development [34]. From an immunological perspective, pro-inflammatory cytokines such as interleukin 6 (IL-6) and tumor necrosis factor (TNF)-α, generated during atopic responses, can disrupt the blood–brain barrier and exert negative effects on mood and mood regulation. This, in turn, influences neural circuits associated with behavioral functions and elicits neuroimmune responses [35]. Moreover, mast cells, known as major effector cells in AD pathogenesis, can be perinatally activated by various factors, leading to the production of neurotoxins that contribute to the mechanisms underlying ASD [36]. Consequently, these findings highlight the importance of maintaining clinical vigilance when diagnosing AD, enabling early assessment of the potential presence of ASD and timely intervention.

Interestingly, our reverse MR analysis also yielded noteworthy results that BD and AN are causally associated with an elevated risk of developing AD. Subsequently, through an extensive review of the literature, we encountered several clinical cases reporting a history of comorbidity between bipolar disorder and severe AD [37, 38]. Moreover, previous investigations have identified a connection between dietary alterations and disturbances in the skin barrier, leading to the development of atopic skin allergy [39]. In Canine AD research, a study examining its mechanism revealed the up-regulation of genes such as IGHM, IGLL5, CD79B, PIGR, CBS, ASS1, SLPI, and MRRF in the raw food feeding group. These up-regulations enhance innate immunity and reduce oxidative stress in dogs, potentially preventing early onset allergies and immune disorders [40]. This finding may provide insight into the causal relationship between AN and the heightened risk of AD. Moreover, consistent with the results of the MR study by Baurecht et al. [17], we did not find a causal relationship between AD and MDD/anxiety.

Several studies have provided evidence for a significant association between AD and ADHD in children [41]. However, our findings indicate a bidirectional or mutual relationship between AD and ADHD, without a clear causal relationship in terms of temporal order. We speculate that common genetic factors or shared underlying physiological mechanisms, such as inflammation and dysregulation of the immune system, could contribute to the development of both diseases simultaneously, resulting in this bidirectional association. Hypersecretion of inflammatory mediators, including cytokines derived from Th1, Th2, and Th17 cells, may potentially disrupt the maturation process and neural activity of the prefrontal and anterior cingulate cortices, offering a plausible explanation for the coexistence of AD and ADHD [42, 43]. Certainly, considering the more pronounced results obtained from the reverse MR analyses (i.e., ADHD exhibited an increased risk effect to AD), and acknowledging the inherent complexities in establishing causal effects between atopic dermatitis and psychopathology, it is evident that achieving a comprehensive understanding of the relationship between these two conditions may necessitate further research encompassing genetics, physiology, and environmental factors.

## Conclusion

In summary, our study elucidates a multifaceted genetic interplay between AD and psychiatric diseases within a population of European ancestry. Significantly, our findings indicates a causal association between AD and a heightened risk of autism spectrum disorder, and also identifies anorexia nervosa and bipolar disorder as previously unrecognized risk factors for AD in clinical research. These results indicate the significance of addressing and managing AD as a potential approach to the prevention, management, and treatment of psychiatric disorders.

### Supplementary Information

**Additional file 1: Figure S1.** Radial plots to visualize individual outlier single nucleotide polymorphisms (SNPs) in the Mendelian randomization (MR) estimates for association between AD and psychiatric disorders. IVW and MR-Egger outliers (Purple dot) were removed. **Figure S2.** Forest plots of the causal association using two-sample MR analysis methods between atopic dermatitis and psychiatric disorders. Psychiatric disorder including ADHD, AN, Anxiety, ASD, BD, MDD, Schizophrenia and TS as exposures, and atopic dermatitis as outcome. **Figure S3.** Leave-one-out plots of the causal relationships between atopic dermatitis and psychiatric disorders. **Table S1.** Summary of the casual relationships of atopic dermatitis (AD) and psychiatric disease with Mendelian randomization method. **Table S2.** Inferring casual relationships of atopic dermatitis (AD) on psychiatric disease using cML-MA method. **Table S3.** The casual relationships of psychiatric disease and atopic dermatitis (AD) with reverse Mendelian randomization (MR) method. **Table S4.** Inferring casual relationships of psychiatric disease on atopic dermatitis (AD) using cML-MA method. **Table S5.** The horizontal pleiotropy and heterogeneity results in reverse MR. 

## Data Availability

The datasets analyzed in this study are publicly available from the GWAS catalog (https://www.ebi.ac.uk/), PGC (https://www.med.unc.edu/pgc/download-results), and OpenGWAS Consortium (https://gwas.mrcieu.ac.uk/).

## Abbreviations

AD: Atopic dermatitis
ADHD: Attention deficit hyperactivity disorder
AN: Anorexia nervosa
ASD: Autism spectrum disorder
BD: Bipolar disorder
MDD: Major depressive disorder
TS: Tourette syndrome
MR: Mendelian randomization
IVW: Inverse variance weighted
OR: Odds ratio
CI: Confidence interval
cML: Constrained maximum likelihood
MA: Model averaging
