# Supplementary material for: Causal relationships between atopic dermatitis and psychiatric disorders: a bidirectional two-sample Mendelian randomization study
Source: BMC Psychiatry. 2024 Jan 3;24:16. doi: 10.1186/s12888-023-05478-1 (PMC10763185; doi:10.1186/s12888-023-05478-1)
Supplement: Supplementary file 1 — Additional file 1: Figure S1. Radial plots to visualize individual outlier single nucleotide polymorphisms (SNPs) in the Mendelian randomization (MR) estimates for association between AD and psychiatric disorders. IVW and MR-Egger outliers (Purple dot) were removed. Figure S2. Forest plots of the causal association using two-sample MR analysis methods between atopic dermatitis and psychiatric disorders. Psychiatric disorder including ADHD, AN, Anxiety, ASD, BD, MDD, Schizophrenia and TS as exposures, and atopic dermatitis as outcome. Figure S3. Leave-one-out plots of the causal relationships between atopic dermatitis and psychiatric disorders. Table S1. Summary of the casual relationships of atopic dermatitis (AD) and psychiatric disease with Mendelian randomization method. Table S2. Inferring casual relationships of atopic dermatitis (AD) on psychiatric disease using cML-MA method. Table S3. The casual relationships of psychiatric disease and atopic dermatitis (AD) with reverse Mendelian randomization (MR) method. Table S4. Inferring casual relationships of psychiatric disease on atopic dermatitis (AD) using cML-MA method. Table S5. The horizontal pleiotropy and heterogeneity results in reverse MR. [file 12888_2023_5478_MOESM1_ESM.docx]

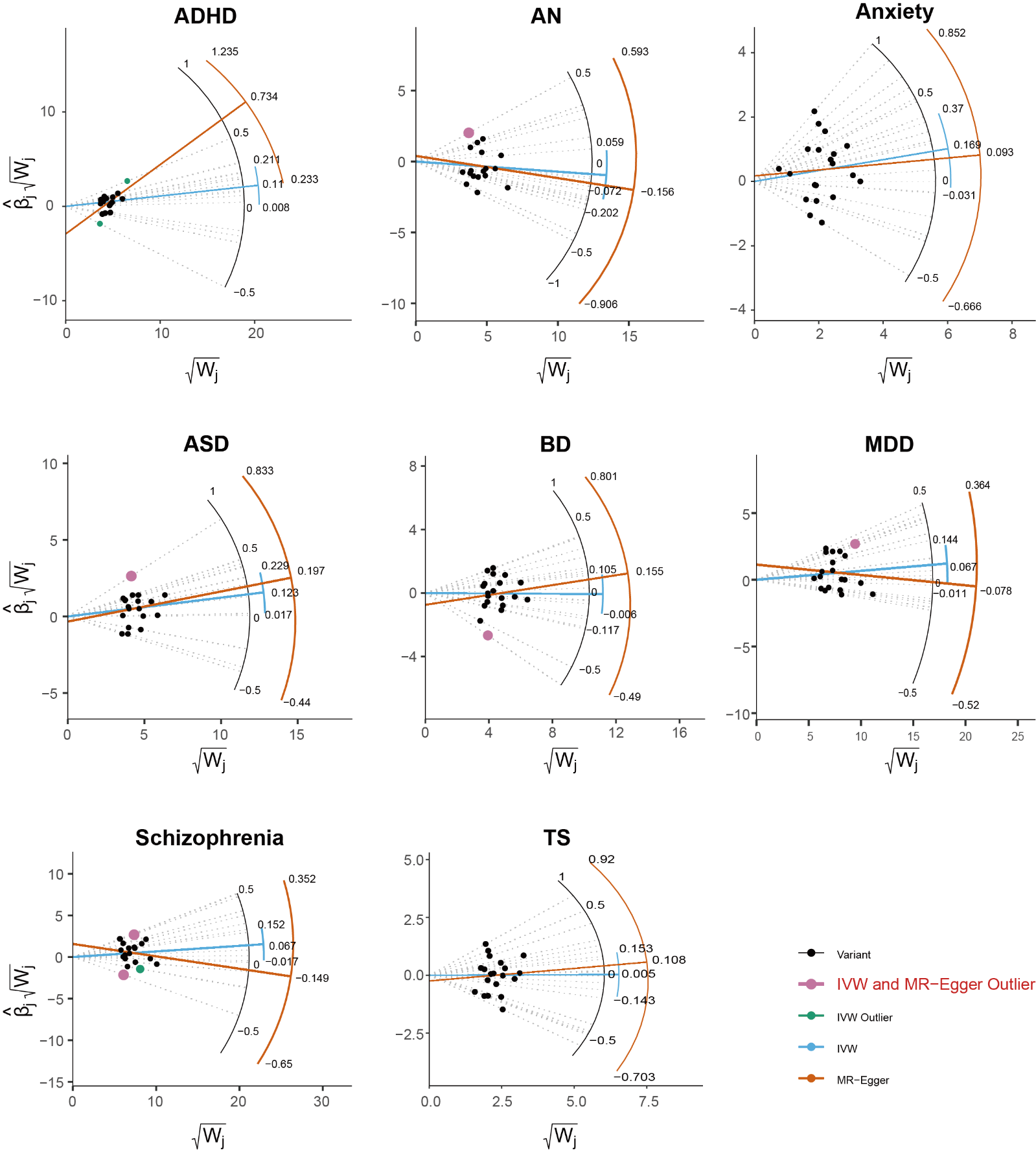


**Figure S1:** Radial plots to visualize individual outlier single nucleotide polymorphisms (SNPs) in the Mendelian randomization (MR) estimates for association between AD and psychiatric disorders. IVW and MR-Egger outliers (Purple dot) were removed.


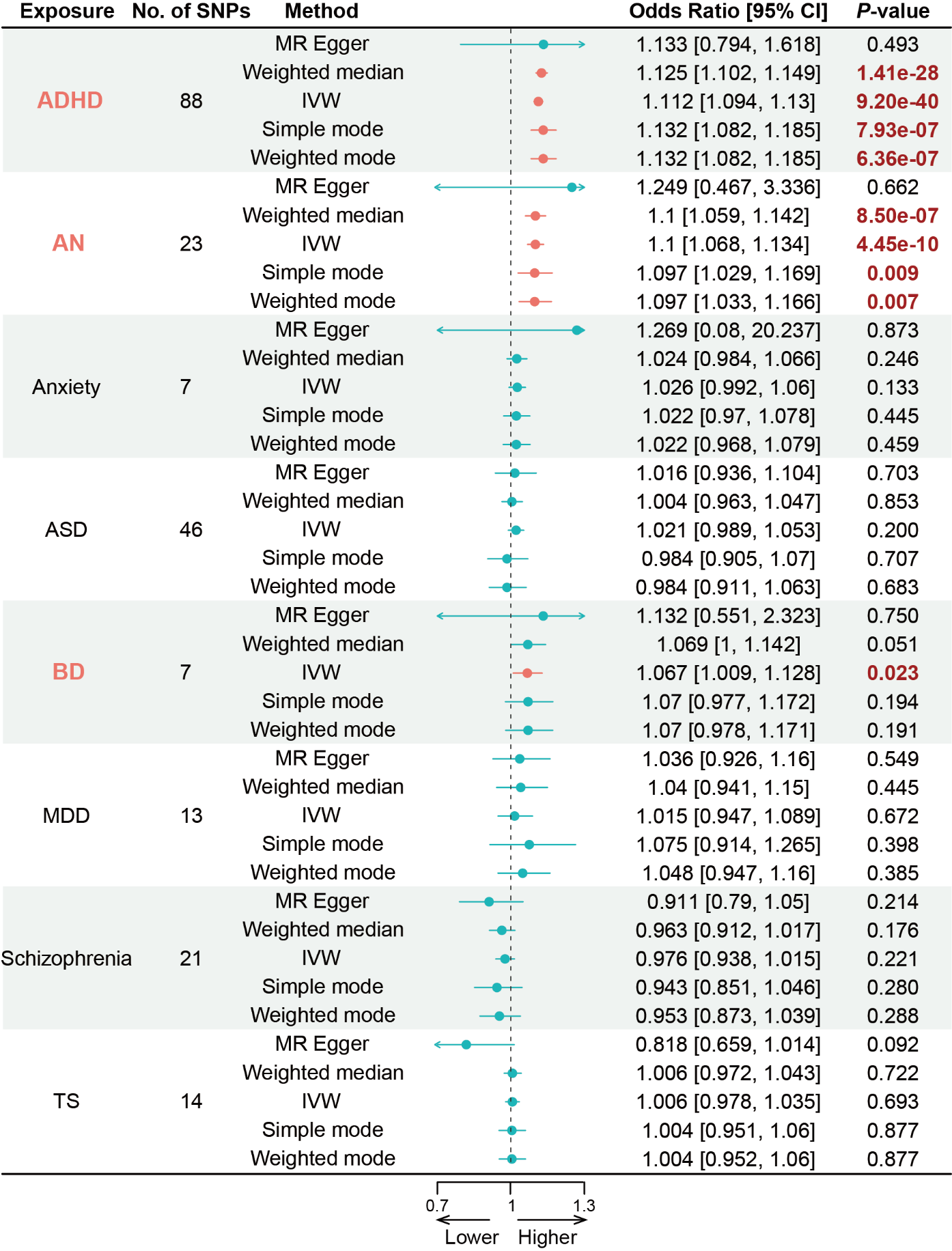


**Figure S2:** Forest plots of the causal association using two-sample MR analysis methods between atopic dermatitis and psychiatric disorders. Psychiatric disorder including ADHD, AN, Anxiety, ASD, BD, MDD, Schizophrenia and TS as exposures, and atopic dermatitis as outcome.


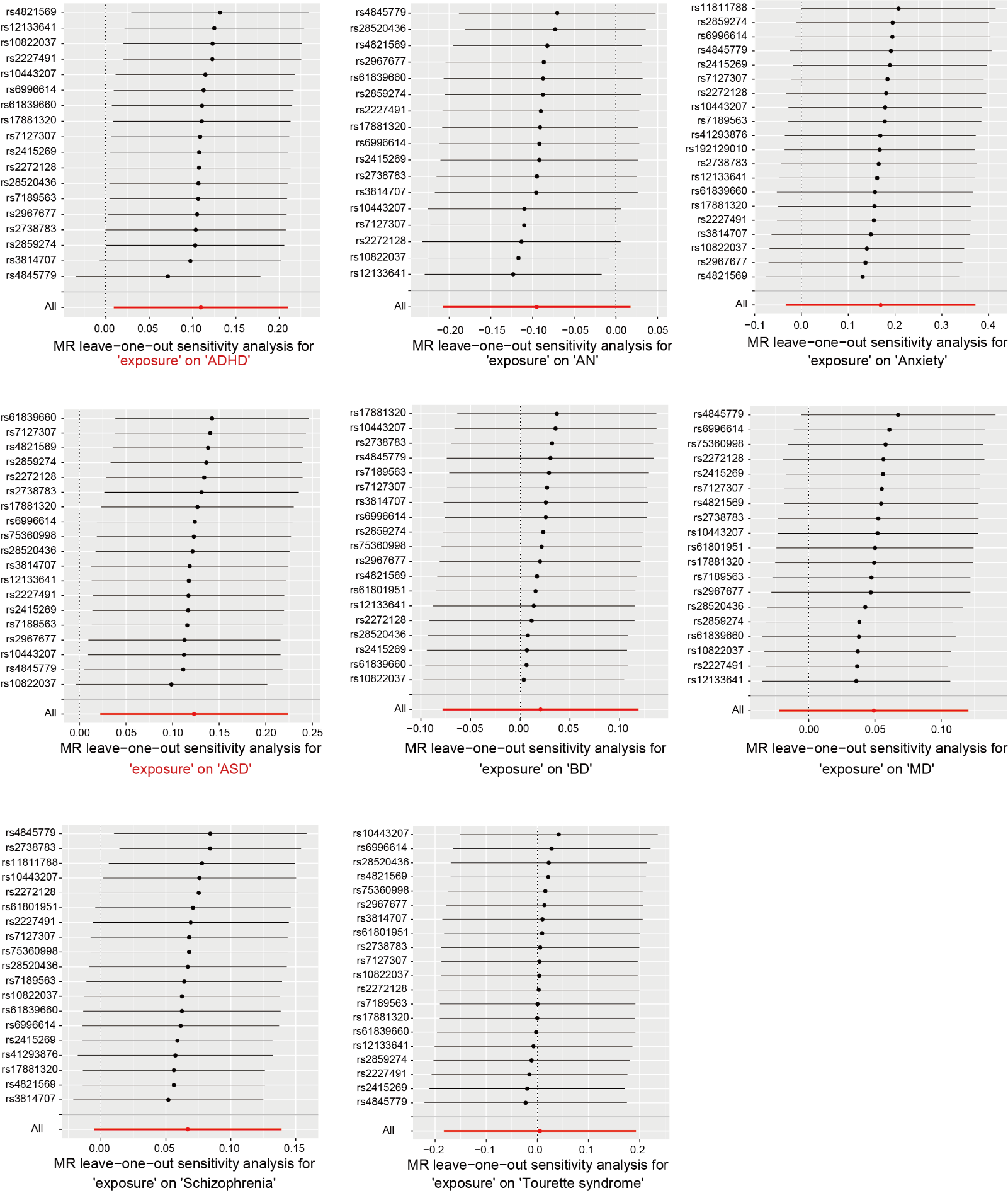


**Figure S3:** Leave-one-out plots of the causal relationships between atopic dermatitis and psychiatric disorders.

**Table S1. Summary of the casual relationships of atopic dermatitis (AD) and psychiatric disease with Mendelian randomization method**

| **Outcome** | **SNP** | **Method** | **Beta** | **SE** | **P-value** |
| --- | --- | --- | --- | --- | --- |
| **ADHD** | 18 | MR Egger | 0.496 | 0.190 | **0.019** |
|  |  | Weighted median | 0.136 | 0.073 | 0.063 |
|  |  | IVW | 0.110 | 0.051 | **0.033** |
|  |  | Simple mode | 0.162 | 0.122 | 0.203 |
|  |  | Weighted mode | 0.155 | 0.122 | 0.223 |
| AN | 17 | MR Egger | -0.359 | 0.209 | 0.107 |
|  |  | Weighted median | -0.145 | 0.075 | 0.054 |
|  |  | IVW | -0.095 | 0.057 | 0.098 |
|  |  | Simple mode | -0.210 | 0.138 | 0.150 |
|  |  | Weighted mode | -0.214 | 0.128 | 0.114 |
| Anxiety | 20 | MR Egger | 0.213 | 0.420 | 0.617 |
|  |  | Weighted median | 0.183 | 0.144 | 0.203 |
|  |  | IVW | 0.169 | 0.103 | 0.101 |
|  |  | Simple mode | 0.265 | 0.235 | 0.275 |
|  |  | Weighted mode | 0.139 | 0.224 | 0.542 |
| **ASD** | 19 | MR Egger | 0.108 | 0.171 | 0.537 |
|  |  | Weighted median | 0.164 | 0.074 | **0.026** |
|  |  | IVW | 0.123 | 0.051 | **0.016** |
|  |  | Simple mode | 0.216 | 0.128 | 0.108 |
|  |  | Weighted mode | 0.198 | 0.116 | 0.107 |
| BD | 19 | MR Egger | 0.075 | 0.161 | 0.647 |
|  |  | Weighted median | -0.020 | 0.071 | 0.775 |
|  |  | IVW | 0.020 | 0.050 | 0.685 |
|  |  | Simple mode | -0.032 | 0.131 | 0.811 |
|  |  | Weighted mode | -0.035 | 0.110 | 0.757 |
| MDD | 19 | MR Egger | -0.032 | 0.111 | 0.777 |
|  |  | Weighted median | 0.002 | 0.046 | 0.970 |
|  |  | IVW | 0.049 | 0.036 | 0.177 |
|  |  | Simple mode | -0.045 | 0.094 | 0.636 |
|  |  | Weighted mode | -0.055 | 0.073 | 0.462 |
| Schizophrenia | 19 | MR Egger | -0.034 | 0.114 | 0.766 |
|  |  | Weighted median | 0.040 | 0.047 | 0.391 |
|  |  | IVW | 0.067 | 0.037 | 0.070 |
|  |  | Simple mode | 0.040 | 0.103 | 0.704 |
|  |  | Weighted mode | -0.026 | 0.101 | 0.800 |
| TS | 20 | MR Egger | -0.240 | 0.319 | 0.462 |
|  |  | Weighted median | 0.027 | 0.129 | 0.833 |
|  |  | IVW | 0.005 | 0.096 | 0.959 |
|  |  | Simple mode | 0.058 | 0.224 | 0.799 |
|  |  | Weighted mode | 0.054 | 0.200 | 0.790 |

**Table S2. Inferring casual relationships of atopic dermatitis (AD) on psychiatric disease using cML-MA method**

| Outcome  Results | **ADHD** | | **AN** | **Anxiety** | **ASD** | **BD** | **MDD** | **Schizophrenia** | **TS** |
| --- | --- | --- | --- | --- | --- | --- | --- | --- | --- |
| Theta | 0.091 | -0.097 | | 0.169 | 0.123 | 0.021 | 0.050 | 0.005 | 0.006 |
| SE | 0.052 | 0.053 | | 0.104 | 0.052 | 0.051 | 0.030 | 0.022 | 0.096 |
| P-value | 0.077 | 0.067 | | 0.104 | **0.018** | 0.678 | 0.101 | 0.829 | 0.950 |

**Table S3. The casual relationships of psychiatric disease and atopic dermatitis (AD) with reverse Mendelian randomization (MR) method**

| **Exposure** | **SNP** | **Method** | **Beta** | **SE** | **P-value** |
| --- | --- | --- | --- | --- | --- |
| **ADHD** | 88 | MR Egger | 0.125 | 0.182 | 0.493 |
|  |  | Weighted median | 0.118 | 0.011 | **1.41e-28** |
|  |  | IVW | 0.106 | 0.008 | **9.20e-40** |
|  |  | Simple mode | 0.124 | 0.023 | **7.93e-07** |
|  |  | Weighted mode | 0.124 | 0.023 | **6.36e-07** |
| **AN** | 23 | MR Egger | 0.222 | 0.501 | 0.662 |
|  |  | Weighted median | 0.095 | 0.019 | **8.50e-07** |
|  |  | IVW | 0.095 | 0.015 | **4.45e-10** |
|  |  | Simple mode | 0.092 | 0.032 | **0.009** |
|  |  | Weighted mode | 0.093 | 0.031 | **0.007** |
| Anxiety | 7 | MR Egger | 0.238 | 1.413 | 0.873 |
|  |  | Weighted median | 0.024 | 0.02 | 0.246 |
|  |  | IVW | 0.025 | 0.017 | 0.133 |
|  |  | Simple mode | 0.022 | 0.027 | 0.445 |
|  |  | Weighted mode | 0.022 | 0.028 | 0.459 |
| ASD | 46 | MR Egger | 0.016 | 0.042 | 0.703 |
|  |  | Weighted median | 0.004 | 0.021 | 0.853 |
|  |  | IVW | 0.021 | 0.016 | 0.200 |
|  |  | Simple mode | -0.016 | 0.043 | 0.707 |
|  |  | Weighted mode | -0.016 | 0.039 | 0.683 |
| **BD** | 7 | MR Egger | 0.124 | 0.367 | 0.750 |
|  |  | Weighted median | 0.066 | 0.034 | 0.051 |
|  |  | IVW | 0.065 | 0.028 | **0.023** |
|  |  | Simple mode | 0.068 | 0.046 | 0.194 |
|  |  | Weighted mode | 0.068 | 0.046 | 0.191 |
| MDD | 13 | MR Egger | 0.036 | 0.057 | 0.549 |
|  |  | Weighted median | 0.039 | 0.051 | 0.445 |
|  |  | IVW | 0.015 | 0.036 | 0.672 |
|  |  | Simple mode | 0.073 | 0.083 | 0.398 |
|  |  | Weighted mode | 0.047 | 0.052 | 0.385 |
| Schizophrenia | 21 | MR Egger | -0.093 | 0.073 | 0.214 |
|  |  | Weighted median | -0.038 | 0.028 | 0.176 |
|  |  | IVW | -0.025 | 0.02 | 0.221 |
|  |  | Simple mode | -0.058 | 0.053 | 0.280 |
|  |  | Weighted mode | -0.048 | 0.044 | 0.288 |
| TS | 14 | MR Egger | -0.201 | 0.11 | 0.092 |
|  |  | Weighted median | 0.006 | 0.018 | 0.722 |
|  |  | IVW | 0.006 | 0.014 | 0.693 |
|  |  | Simple mode | 0.004 | 0.028 | 0.877 |
|  |  | Weighted mode | 0.004 | 0.028 | 0.877 |

**Table S4. Inferring casual relationships of psychiatric disease on atopic dermatitis (AD) using cML-MA method**

| Exposure  Results | **ADHD** | **AN** | **Anxiety** | **ASD** | **BD** | **MDD** | **Schizophrenia** | **TS** |
| --- | --- | --- | --- | --- | --- | --- | --- | --- |
| Theta | 0.106 | 0.096 | 0.025 | 0.018 | 0.065 | 0.025 | -0.025 | 0.006 |
| SE | 0.008 | 0.016 | 0.017 | 0.015 | 0.029 | 0.035 | 0.019 | 0.014 |
| P-value | **3.64e-38** | **7.83e-10** | 0.135 | 0.250 | **0.024** | 0.462 | 0.197 | 0.691 |

**Table S5. The horizontal pleiotropy and heterogeneity results in reverse MR**

| **Exposure** | **Horizontal pleiotropy** | | | **Heterogeneity** | | | |
| --- | --- | --- | --- | --- | --- | --- | --- |
|  |  |  |  | **MR Egger** | | **IVW** | |
|  | **Intercept** | **SE** | **P-value** | **Q** | **P-value** | **Q** | **P-value** |
| ADHD | -0.002 | 0.018 | 0.917 | 16.193 | 1 | 16.204 | 1 |
| AN | -0.020 | 0.078 | 0.803 | 2.350 | 1 | 2.414 | 1 |
| Anxiety | -0.032 | 0.216 | 0.886 | 0.073 | 1 | 0.095 | 1 |
| ASD | 0.0004 | 0.004 | 0.911 | 42.612 | 0.531 | 42.625 | 0.573 |
| BD | -0.006 | 0.035 | 0.878 | 0.196 | 0.999 | 0.222 | 1 |
| MDD | -0.002 | 0.004 | 0.659 | 6.792 | 0.816 | 6.998 | 0.858 |
| Schizophrenia | 0.006 | 0.007 | 0.337 | 20.998 | 0.337 | 22.068 | 0.337 |
| TS | 0.029 | 0.015 | 0.082 | 1.224 | 1 | 4.838 | 0.979 |

IVW: Inverse variance weighted

ADHD: Attention deficit hyperactivity disorder

AN: Anorexia nervosa

ASD: Autism spectrum disorder

BD: Bipolar disorder

MDD: Major depressive disorder

TS: Tourette syndrome

cML-MA: constrained maximum likelihood and model averaging
